# Comparison of mol­ecular structures of *cis*-bis­[8-(di­methyl­phosphan­yl)quinoline]­nickel(II) and -platinum(II) complex cations

**DOI:** 10.1107/S2056989020014437

**Published:** 2020-11-06

**Authors:** Masatoshi Mori, Takayoshi Suzuki

**Affiliations:** a Graduate School of Natural Science and Technology, Okayama University, Okayama, 700-8530, Japan; b Research Institute for Interdisciplinary Science, Okayama University, Okayama, 700-8530, Japan

**Keywords:** 8-quinolylphosphane, asymmetrical bidentate ligand, square-planar coordination, tetra­hedral distortion, *trans* influence, crystal structure

## Abstract

The crystal structures of the complexes (*SP*-4-2)-*cis*-bis­[8-(di­methyl­phosphan­yl)quinoline-κ^2^ *N*,*P*]nickel(II) bis­(perchlorate) nitro­methane monosolvate, [Ni(C_11_H_12_NP)_2_](ClO_4_)_2_·CH_3_NO_2_ (**1**), and (*SP*-4-2)-*cis*-bis­[8-(di­methyl­phos­phan­yl)quinoline-κ^2^ *N*,*P*]platinum(II) bis­(tetra­fluoro­borate) aceto­nitrile monosolvate, [Pt(C_11_H_12_NP)_2_](BF_4_)_2_·C_2_H_3_N (**2**), are reported. In both complex cations, two phosphanyl­quinolines act as bidentate *P,N*-donating chelate ligands and form the mutually *cis* configuration in the square-planar coordination geometry. The strong *trans* influence of the di­methyl­phosphanyl donor group is confirmed by the Ni—N bond lengths in **1**, 1.970 (2) and 1.982 (2) Å and, the Pt—N bond lengths of **2**, 2.123 (4) and 2.132 (4) Å, which are relatively long as compared to those in the analogous 8-(di­phenyl­phosphan­yl)quinoline complexes. Mutually *cis*-positioned quinoline donor groups would give a severe steric hindrance between their *ortho*-H atoms. In order to reduce such a steric congestion, the Ni^II^ complex in **1** shows a tetra­hedral distortion of the coordination geometry, as parameterized by τ_4_ = 0.199 (1)°, while the Pt^II^ complex in **2** exhibits a typical square-planar coordination geometry [τ_4_ = 0.014 (1)°] with a large bending deformation of the ideally planar Me_2_Pqn chelate planes. In the crystal structure of **2**, three F atoms of one of the BF_4_^−^ anions are disordered over two sets of positions with refined occupancies of 0.573 (10) and 0.427 (10).

## Abstract 2

Owing to the intra­molecular steric hindrance between the mutually *cis*-positioned 8-(di­methyl­phosphan­yl)quinoline (Me_2_Pqn) ligands, the nickel(II) complex of *cis*-[Ni(Me_2_Pqn)_2_]^2+^ shows a remarkable tetra­hedral distortion of the Ni^II^ coordination geometry. In contrast, the corresponding platinum(II) complex of *cis*-[Pt(Me_2_Pqn)_2_]^2+^ exhibits a typical square-planar Pt^II^ coordination center, but large envelope-type distortions of the five-membered chelate planes are observed.

## Chemical context   

8-Quinolylphosphanes are an intriguing class of ligands because they form a planar asymmetrical five-membered chelate ring *via* coordination through a phosphane-P atom having a strong σ-donating character and an imine-N atom incorporated in a π-conjugated quinoline ring (Salem & Wild, 1992 ▸; Scattolin *et al.*, 2017 ▸; Cai *et al.*, 2018 ▸). The electronic properties of these ligands, in particular their π-bonding nature, may stabilize unusual electronic states of their transition-metal complexes (Suzuki *et al.*, 1995 ▸; Hashimoto *et al.*, 2010 ▸; Hopkins *et al.*, 2019 ▸). In addition, the steric requirement from the planar quinoline moiety often has a significant influence on the properties of their metal complexes. For example, the nickel(II), palladium(II) and platinum(II) complexes containing two 8-(di­phenyl­phosphan­yl)quinoline (Ph_2_Pqn) in the *cis(P,P)* configuration exhibit a severe distortion of the square-planar coordination geometry around *M* ^II^ (*M* = Ni, Pd or Pt; Suzuki, 2004 ▸; Hashimoto *et al.*, 2010 ▸; Mori *et al.*, 2020 ▸). The di­methyl­phosphanyl analogue, 8-(di­methyl­phosphan­yl)quinoline (Me_2_Pqn), is an inter­esting derivative, because it would give a stronger *trans* influence, which could affect the steric congestion between the intra­molecular ligands. However, the transition-metal complexes bearing Me_2_Pqn are limited to only those listed in section 4: *Database survey*, all of which were reported by our group. In 1995 we reported the preparation and crystal structure of (*SP*-4-2)-[Pd(Me_2_Pqn)_2_](BF_4_)_2_ (Suzuki *et al.*, 1995 ▸), but the crystal structures of the corresponding Ni^II^ and Pt^II^ complexes were not compared.
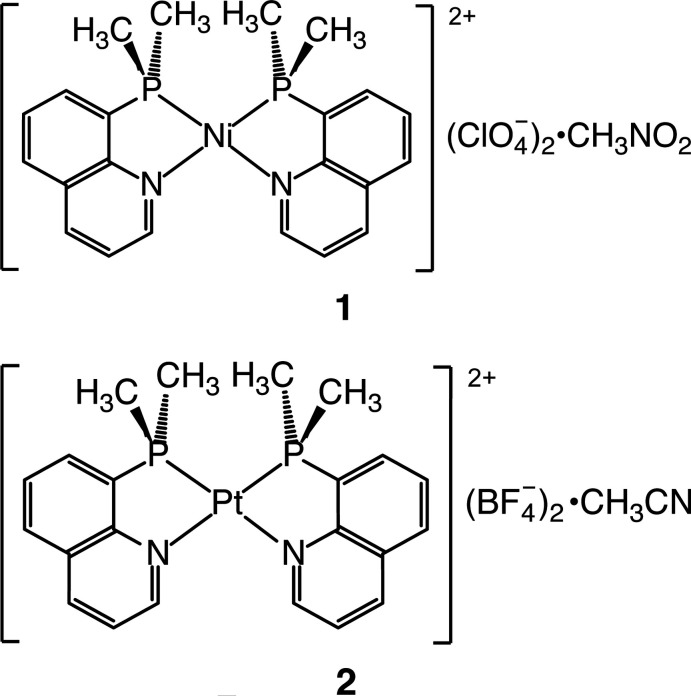


## Structural commentary   

A red block-shaped crystal of the Ni^II^ complex, [Ni(Me_2_Pqn)_2_](ClO_4_)_2_·CH_3_NO_2_ (**1**), recrystallized from nitro­methane/diisopropyl ether and a colorless platelet crystal of the Pt^II^ complex, [Pt(Me_2_Pqn)_2_](BF_4_)_2_·CH_3_CN (**2**), recrystallized from aceto­nitrile/diisopropyl ether were used for the X-ray diffraction analysis.

In the crystal structure of **2**, three F atoms of one of the BF_4_^−^ anions show disorder over two sets of positions: (F2*A*, F3*A* and F4*A*) and (F2*B*, F3*B* and F4*B*). The occupancy parameters of these atoms were refined with suitable restrictions and found to be 0.573 (10) and 0.427 (10) for the *A*-set atoms and the *B*-set atoms, respectively.

**Figure 1:**
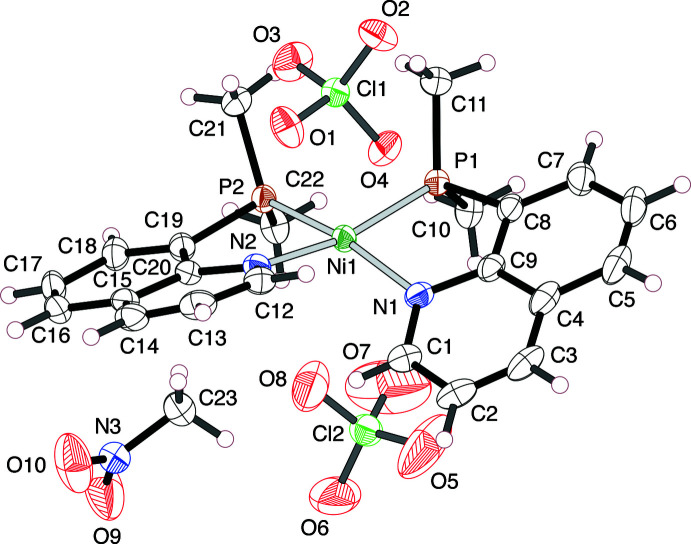
An ellipsoid plot of the mol­ecular structures in [Ni(Me_2_Pqn)_2_](ClO_4_)_2_·CH_3_NO_2_ (**1**), showing the atom-numbering scheme, with ellipsoids drawn at the 50% probability level.

In both crystals, two Me_2_Pqn ligands coordinate to a metal(II) center in the bidentate κ^2^ *P,N* mode to form a *cis*-isomer of the complex dication, (*SP*-4-2)-[*M*(Me_2_Pqn)_2_]^2+^ (*M* = Ni or Pt), having a roughly square-planar coordination geometry (Figs. 1 ▸ and 2 ▸, Tables 1 ▸ and 2 ▸). For the group 10 metal(II) complexes bearing 8-quinolylphosphanes, it was revealed that most of the bis­(κ^2^ *P,N*)-type complexes have a similar geometrical structure, except for those of the halide complexes (Suzuki, 2004 ▸; Mori *et al.*, 2020 ▸), because the strong *trans* influence of the phosphane donor groups makes the mutually *trans(P,P)* configuration thermodynamically unstable. The Ni—N bond lengths in **1** are 1.970 (2) and 1.982 (2) Å, which are slightly longer than those in [Ni(MePhPqn)_2_](BF_4_)_2_ [MePhPqn = 8-(methyl­phenyl­phosphan­yl)quinoline; 1.954 (3) and 1.977 (3) Å] and [Ni(Ph_2_Pqn)_2_](BF_4_)_2_ [1.954 (6) and 1.949 (5) Å] (Hashimoto *et al.*, 2010 ▸), indicating the *trans* influence becomes stronger in the order of Ph_2_Pqn < MePhPqn < Me_2_Pqn. In the case of Pt^II^ complexes, the Pt—N bond lengths in **2** [2.123 (4) and 2.132 (4) Å] are similarly long, as compared to those in [Pt(Ph_2_Pqn)_2_](ClO_4_)_2_ [2.107 (4) and 2.108 (5) Å; Mori *et al.*, 2020 ▸]. By contrast, the Ni—P bond lengths and the P—Ni—N chelate bite angles are comparable among the complexes **1** [2.1576 (7) and 2.1534 (7) Å; 86.13 (7) and 85.97 (6)°], [Ni(MePhPqn)_2_](BF_4_)_2_ [2.151 (1) and 2.162 (1) Å; 87.4 (1) and 86.6 (1)°] and [Ni(Ph_2_Pqn)_2_](BF_4_)_2_ [2.168 (2) and 2.177 (2) Å; 86.6 (1) and 84.6 (1)°]. The Pt—P bond lengths and the P—Pt—N bite angles in **2** [2.2293 (12) and 2.2365 (12) Å; 82.76 (11) and 81.93 (11)°] are also comparable to those in [Pt(Ph_2_Pqn)_2_](ClO_4_)_2_ [2.2311 (14) and 2.2318 (14) Å; 83.29 (13) and 82.79 (13)°].

**Figure 3:**
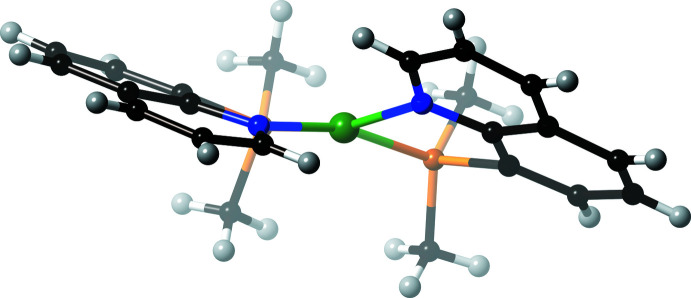
A perspective side view (from one of the NiPN coordination planes) of [Ni(Me_2_Pqn)_2_](ClO_4_)_2_·CH_3_NO_2_ (**1**). Color code: Ni, dark green; P, orange; N, blue; C, black and H, gray.

**Figure 4:**
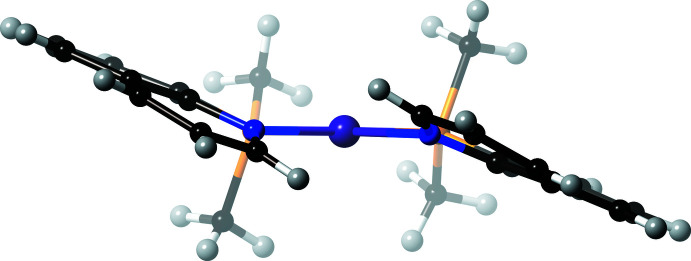
A perspective side view (from one of the PtPN coordination planes) of [Pt(Me_2_Pqn)_2_](BF_4_)_2_·CH_3_CN (**2**). Color code: Pt, purple; P, orange; N, blue; C, black and H, gray.

Comparison of the Ni^II^ complex cation in **1** and the corres­ponding Pt^II^ complex cation in **2** shows an obvious difference in their coordination geometry (Figs. 3 ▸ and 4 ▸). The four-coordinate Ni^II^ center in **1** exhibits a large tetra­hedral distortion, as indicated by the τ_4_ value (Yang *et al.*, 2007 ▸) of 0.199 (1)°. This is due to the steric requirement from the planar quinoline moiety located in the mutually *cis* positions around the Ni^II^ center. In the analogous MePhPqn and Ph_2_Pqn complexes, the τ_4_ values are 0.273 (1)° and 0.189 (2)°, respectively. By contrast, the τ_4_ value of the Pt^II^ complex in **2** is only 0.014 (1)°, indicating a nearly perfect planar coordin­ation geometry around the Pt^II^ center. The corresponding value in [Pt(Ph_2_Pqn)_2_](BF_4_)_2_ is 0.149 (2)° (Mori *et al.*, 2020 ▸). It is obvious that the present planar structure of the Pt^II^ center in **2** is a rare example. In this complex, the inter­ligand steric inter­action expected for the mutually *cis*-positioned quinoline groups could be reduced by envelope-type bending of the planar Me_2_Pqn chelate coordination, that is, the displacement of the Pt^II^ metal center from the ideal plane defined by the chelate ring of 8-quinolylphosphanes. The dihedral angle, *φ*_C_, between the [Pt,P,N] coordination plane and the [PCCN] phosphanyl­quinoline planes in **2** are 21.53 (16) and 24.76 (16)°, and the displacement of the Pt1 atom from the ideal quinoline [C_9_H_6_N] planes is 0.579 (5) and 0.550 (5) Å. The two quinoline planes are nearly parallel, with the dihedral angle between them being only 7.99 (10)°. Such a synchronized bending deformation of two chelate coordination (Fig. 4 ▸) acts to reduce the steric congestion effectively. The corresponding *φ*_C_ values for **1** are 17.44 (9) and 19.76 (9)°, and the dihedral angle between the two quinoline planes is obviously large, at 33.35 (6)°. Inter­estingly, the analogous palladium(II) complex, [Pd(Me_2_Pqn)_2_](BF_4_)_2_, has a τ_4_ value of 0.096 (2)° (Suzuki *et al.*, 1995 ▸), which is in between those of the present Ni^II^ and Pt^II^ complexes.

## Supra­molecular features   

**Figure 5:**
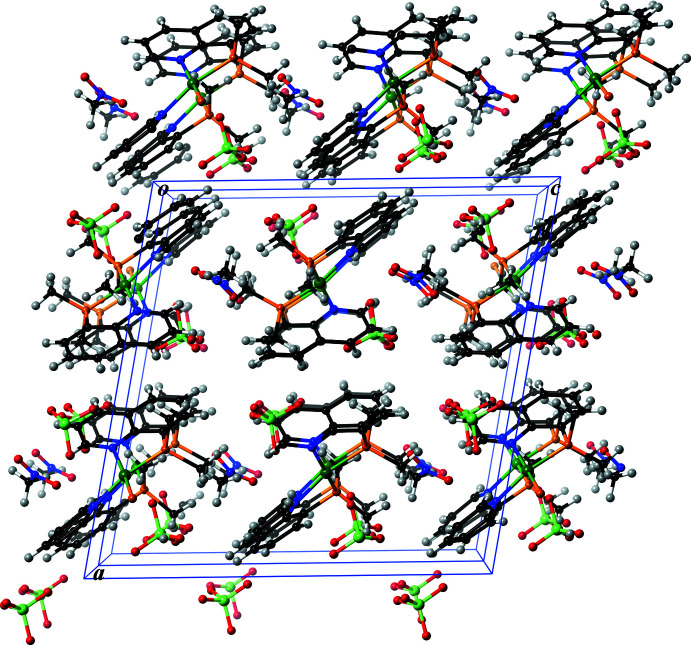
A packing drawing of [Ni(Me_2_Pqn)_2_](ClO_4_)_2_CH_3_NO_2_ (**1**) along the crystallographic *b* axis (two unit cells are shown). Color code: Ni, dark green; Cl, light green; P, orange; O, red; N, blue; C, black and H, gray.

In the crystal structure of **1**, there are two ClO_4_^−^ anions and a CH_3_NO_2_ solvent mol­ecule, in addition to the [Ni(Me_2_Pqn)_2_]^2+^ complex cation in the asymmetric unit. The asymmetric unit of the Pt^II^ complex, **2**, contains a [Pt(Me_2_Pqn)_2_]^2+^ complex cation, two BF_4_^−^ anions (in one of which the positions of three F atoms are disordered) and a CH_3_CN solvent mol­ecule. In the crystal structures of both **1** and **2** (Figs. 5 ▸ and 6 ▸, respectively), no remarkable inter­molecular stacking or hydrogen-bonding inter­actions are observed.

## Database survey   

Metal complexes containing Me_2_Pqn have been reported by us, *e.g*., *cis*-[Pd(Me_2_Pqn)_2_](BF_4_)_2_ (refcode ZIFPUZ in the CSD database, version 5.41, last update May 2020; Groom *et al.*, 2016 ▸) and [Pd_2_Cl_2_(Me_2_Pqn)_2_] (ZIFQAG; Suzuki *et al.*, 1995 ▸), [Cu(Me_2_Pqn)_2_]PF_6_ (OZILAL; Suzuki *et al.*, 2011 ▸), [Ru(bpy)_3–*n*_(Me_2_Pqn)_*n*_](PF_6_)_2_ (bpy = 2,2′-bi­pyridine; HUTRIV, HUTPCB, HUTPUH and HUTQAO; Suzuki *et al.*, 2003 ▸), and [Pt(ppy)(Me_2_Pqn)]BF_4_ (ppy = 2-(2′-pyrid­yl)phenyl; Mori & Suzuki, 2020 ▸). Some of the related bis­(8-quinolylphosphanes) complexes are: [Ni(Ph_2_Pqn)_2_](BF_4_)_*n*_ (*n* = 1 or 2; BUGDAJ, BUGDEN and BUGDOX) and [Ni(MePhPqn)_2_](BF_4_)_2_ (BUGDIR; Hashimoto *et al.*, 2010 ▸), [Pd(Ph_2_Pqn)_2_]*X*_2_ (*X*_2_ = Cl_2_, Br_2_ or ClBF_4_; FERZOS, FERZUY and FESBAH; Suzuki, 2004 ▸), [Cu(Ph_2_Pqn)_2_]BF_4_ (OZILEP and OZILEP01; Suzuki *et al.*, 2011 ▸) and [Cu(Ph_2_Pqn)_2_]PF_6_ (NOPNIQ; Tsukuda *et al.*, 2009 ▸).

## Synthesis and crystallization   

The ligand, Me_2_Pqn, and the nickel(II) complexes, [Ni(Me_2_Pqn)_2_](ClO_4_)_2_, were prepared according to the method reported previously (Suzuki *et al.*, 1995 ▸). Single crystals of **1** suitable for an X-ray diffraction study were obtained by recrystallization from nitro­methane by diffusion of diisopropyl ether. The platinum(II) complex, [Pt(Me_2_Pqn)_2_](BF_4_)_2_, was prepared by the following method. A methanol (5 ml) solution of Me_2_Pqn (0.76 mmol) was added dropwise with stirring to a di­chloro­methane solution (10 ml) of [PtCl_2_(EtCN)_2_] (0.105 g, 0.278 mmol), and the mixture was stirred for 24 h at room temperature. After removal of the resulting precipitate, the filtrate was concentrated to *ca* 5 ml using a rotary evaporator. A large excess amount of a methanol solution of NaBF_4_ was added, and the mixture was stirred for 1 h at room temperature. The resulting pale-yellow precipitate was collected by filtration, washed with water (5 ml) and diethyl ether (10 ml), and dried *in vacuo*. Colorless platelet-shaped crystals of [Pt(Me_2_Pqn)_2_](BF_4_)_2_·CH_3_CN (**2**) were obtained by recrystallization from an aceto­nitrile solution by diffusion of diisopropyl ether. Yield: 0.126 g (61%). Analysis calculated for C_24_H_27_B_2_F_8_N_3_P_2_Pt: C, 35.37; H, 3.24; N, 3.75%. Found (after completely drying): C, 35.39; H, 2.89; N, 3.74%.

## Refinement   

Crystal data, data collection and structure refinement details are summarized in Table 3 ▸. All H atoms were refined using a riding model, with C—H = 0.95 (aromatic) or 0.98 (meth­yl) Å and *U*_iso_(H) = 1.2*U*_eq_(C). In the analysis of **2**, two sets of F atoms for one of the two BF_4_^−^ anions were introduced as positionally disordered atoms, and their occupation parameters were refined with suitable restrictions [the final major:minor occupancy ratio was 0.573 (10):0.427 (10)].

## Supplementary Material

Crystal structure: contains datablock(s) 1, 2, global. DOI: 10.1107/S2056989020014437/pk2650sup1.cif

Structure factors: contains datablock(s) 1. DOI: 10.1107/S2056989020014437/pk26501sup2.hkl

Structure factors: contains datablock(s) 2. DOI: 10.1107/S2056989020014437/pk26502sup3.hkl

CCDC references: 2041421, 2041420

Additional supporting information:  crystallographic information; 3D view; checkCIF report

## Figures and Tables

**Figure 2:**
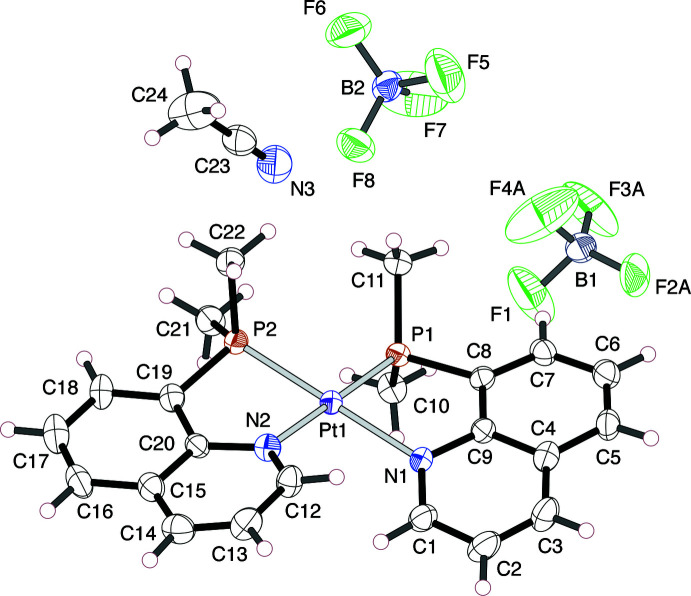
An ellipsoid plot of the mol­ecular structures in [Pt(Me_2_Pqn)_2_](BF_4_)_2_·CH_3_CN (**2**), showing the atom-numbering scheme, with ellipsoids drawn at the 50% probability level. The minor component atoms (F2*B*, F3*B* and F4*B*) of the positionally disordered F atoms are omitted for clarity.

**Figure 6:**
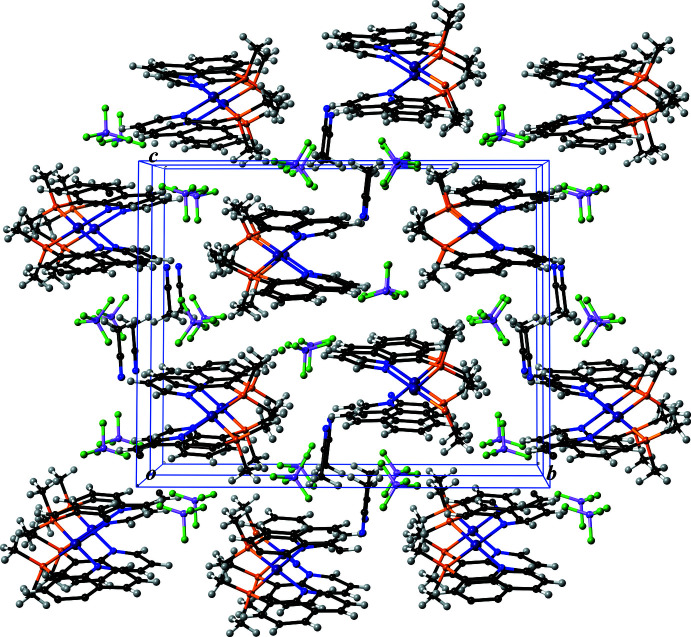
A packing drawing of [Pt(Me_2_Pqn)_2_](BF_4_)_2_·CH_3_CN (**2**) along the crystallographic *a* axis (two unit cells are shown). Color code: Pt, purple; P, orange; F, green; N, blue; C, black; B, pale purple and H, gray.

**Table 1:** Selected geometric parameters (Å, °) for **1** [Chem scheme1]

Ni1—N1	1.970 (2)	Ni1—P2	2.1534 (7)
Ni1—N2	1.982 (2)	Ni1—P1	2.1576 (7)
			
N1—Ni1—N2	97.01 (9)	N1—Ni1—P1	86.13 (7)
N1—Ni1—P2	166.27 (7)	N2—Ni1—P1	165.68 (6)
N2—Ni1—P2	85.97 (6)	P2—Ni1—P1	94.29 (3)
			
Ni1—P1—C8—C9	14.54 (19)	Ni1—P2—C19—C20	18.86 (19)
Ni1—N1—C9—C8	−11.8 (3)	Ni1—N2—C20—C19	−9.7 (3)

**Table 2:** Selected geometric parameters (Å, °) for **2** [Chem scheme1]

Pt1—N1	2.123 (4)	Pt1—P2	2.2293 (12)
Pt1—N2	2.132 (4)	Pt1—P1	2.2365 (12)
			
N1—Pt1—N2	97.13 (15)	N1—Pt1—P1	82.76 (11)
N1—Pt1—P2	178.51 (11)	N2—Pt1—P1	179.55 (11)
N2—Pt1—P2	81.93 (11)	P2—Pt1—P1	98.17 (4)
			
Pt1—P1—C8—C9	15.5 (3)	Pt1—P2—C19—C20	21.2 (4)
Pt1—N1—C9—C8	−18.9 (5)	Pt1—N2—C20—C19	−19.0 (5)

**Table 3:** Experimental details

	**1**	**2**
Crystal data		
Chemical formula	[Ni(C_11_H_12_NP)_2_](ClO_4_)_2_·CH_3_NO_2_	[Pt(C_11_H_12_NP)_2_](BF_4_)_2_·C_2_H_3_N
*M*_r_	697.02	788.13
Crystal system, space group	Monoclinic, *P*2_1_/*c*	Monoclinic, *P*2_1_/*n*
Temperature (K)	188	188
*a*, *b*, *c* (Å)	17.8114 (13), 8.9398 (6), 18.0245 (14)	7.9102 (3), 21.0833 (5), 16.7519 (4)
β (°)	100.524 (3)	95.3931 (11)
*V* (Å^3^)	2821.8 (4)	2781.42 (15)
*Z*	4	4
Radiation type	Mo *K*α	Mo *K*α
μ (mm^−1^)	1.05	5.23
Crystal size (mm)	0.70 × 0.50 × 0.20	0.30 × 0.30 × 0.10

Data collection		
Diffractometer	Rigaku R-AXIS RAPID	Rigaku R-AXIS RAPID
Absorption correction	Multi-scan (*ABSCOR*; Rigaku, 1995 ▸)	Numerical (*NUMABS*; Rigaku, 1999 ▸)
*T*_min_, *T*_max_	0.379, 0.811	0.338, 0.594
No. of measured, independent and observed [*I* > 2σ(*I*)] reflections	26671, 6459, 5440	43967, 6350, 5435
*R*_int_	0.041	0.042
(sin θ/λ)_max_ (Å^−1^)	0.649	0.649

Refinement		
*R*[*F* ^2^ > 2σ(*F* ^2^)], *wR*(*F* ^2^), *S*	0.043, 0.117, 1.05	0.033, 0.084, 1.07
No. of reflections	6459	6350
No. of parameters	370	366
H-atom treatment	H-atom parameters constrained	H-atom parameters constrained
Δρ_max_, Δρ_min_ (e Å^−3^)	1.03, −0.49	1.42, −1.45

Computer programs: RAPID AUTO (Rigaku, 1998 ▸), *CrystalStructure* (Rigaku, 2010 ▸), *SIR2011* (Burla *et al.*, 2012 ▸), *SHELXL2013* (Sheldrick, 2015 ▸) and *CrystalMaker* (CrystalMaker Software, 2017 ▸).

## supplementary crystallographic information

### (SP-4-2)-cis-Bis[8-(dimethylphosphanyl)quinoline-κ2N,P]nickel(II) bis(perchlorate) nitromethane monosolvate (1) Crystal data

**Table:**

[Ni(C_11_H_12_NP)_2_](ClO_4_)_2_·CH_3_NO_2_	*F*(000) = 1432
*M**_r_* = 697.02	*D*_x_ = 1.641 Mg m^−^^3^
Monoclinic, *P*2_1_/*c*	Mo *K*α radiation, λ = 0.71075 Å
*a* = 17.8114 (13) Å	Cell parameters from 20058 reflections
*b* = 8.9398 (6) Å	θ = 3.2–27.6°
*c* = 18.0245 (14) Å	µ = 1.05 mm^−^^1^
β = 100.524 (3)°	*T* = 188 K
*V* = 2821.8 (4) Å^3^	Block, red
*Z* = 4	0.70 × 0.50 × 0.20 mm

### (SP-4-2)-cis-Bis[8-(dimethylphosphanyl)quinoline-κ2N,P]nickel(II) bis(perchlorate) nitromethane monosolvate (1) Data collection

**Table:**

Rigaku R-AXIS RAPID diffractometer	6459 independent reflections
Radiation source: fine-focus sealed tube	5440 reflections with *I* > 2σ(*I*)
Detector resolution: 10.000 pixels mm^-1^	*R*_int_ = 0.041
ω scans	θ_max_ = 27.5°, θ_min_ = 3.2°
Absorption correction: multi-scan (ABSCOR; Rigaku, 1995)	*h* = −23→23
*T*_min_ = 0.379, *T*_max_ = 0.811	*k* = −11→10
26671 measured reflections	*l* = −23→23

### (SP-4-2)-cis-Bis[8-(dimethylphosphanyl)quinoline-κ2N,P]nickel(II) bis(perchlorate) nitromethane monosolvate (1) Refinement

**Table:**

Refinement on *F*^2^	Primary atom site location: structure-invariant direct methods
Least-squares matrix: full	Secondary atom site location: difference Fourier map
*R*[*F*^2^ > 2σ(*F*^2^)] = 0.043	Hydrogen site location: inferred from neighbouring sites
*wR*(*F*^2^) = 0.117	H-atom parameters constrained
*S* = 1.05	*w* = 1/[σ^2^(*F*_o_^2^) + (0.0596*P*)^2^ + 2.7492*P*] where *P* = (*F*_o_^2^ + 2*F*_c_^2^)/3
6459 reflections	(Δ/σ)_max_ = 0.001
370 parameters	Δρ_max_ = 1.03 e Å^−^^3^
0 restraints	Δρ_min_ = −0.49 e Å^−^^3^

### (SP-4-2)-cis-Bis[8-(dimethylphosphanyl)quinoline-κ2N,P]nickel(II) bis(perchlorate) nitromethane monosolvate (1) Special details

**Table:**

Geometry. All esds (except the esd in the dihedral angle between two l.s. planes) are estimated using the full covariance matrix. The cell esds are taken into account individually in the estimation of esds in distances, angles and torsion angles; correlations between esds in cell parameters are only used when they are defined by crystal symmetry. An approximate (isotropic) treatment of cell esds is used for estimating esds involving l.s. planes.

### (SP-4-2)-cis-Bis[8-(dimethylphosphanyl)quinoline-κ2N,P]nickel(II) bis(perchlorate) nitromethane monosolvate (1) Fractional atomic coordinates and isotropic or equivalent isotropic displacement parameters (Å^2^)

**Table:**

	*x*	*y*	*z*	*U*_iso_*/*U*_eq_	
Ni1	0.74556 (2)	0.21692 (3)	0.05020 (2)	0.02369 (10)	
Cl1	0.60962 (3)	0.03126 (7)	−0.11398 (3)	0.02893 (15)	
Cl2	0.90376 (4)	0.47701 (8)	0.15809 (4)	0.03678 (16)	
P1	0.79934 (4)	0.00551 (7)	0.08449 (4)	0.02582 (15)	
P2	0.68019 (4)	0.22150 (7)	0.13957 (3)	0.02357 (14)	
O1	0.57753 (13)	0.1576 (2)	−0.15803 (13)	0.0477 (5)	
O2	0.59943 (13)	−0.1010 (2)	−0.15961 (11)	0.0409 (5)	
O3	0.57362 (13)	0.0136 (3)	−0.04995 (12)	0.0495 (6)	
O4	0.68979 (11)	0.0582 (3)	−0.08854 (13)	0.0475 (5)	
O5	0.94703 (18)	0.4498 (6)	0.1033 (2)	0.1249 (18)	
O6	0.9264 (2)	0.6110 (4)	0.1969 (2)	0.1026 (13)	
O7	0.9117 (3)	0.3654 (5)	0.2115 (3)	0.156 (2)	
O8	0.82342 (15)	0.4791 (4)	0.12503 (17)	0.0726 (8)	
O9	0.77720 (18)	0.6729 (4)	−0.1337 (2)	0.1098 (15)	
O10	0.70736 (19)	0.6516 (3)	−0.24168 (17)	0.0800 (10)	
N1	0.82335 (12)	0.2389 (2)	−0.01392 (12)	0.0267 (4)	
N2	0.67687 (12)	0.3788 (2)	0.00323 (11)	0.0251 (4)	
N3	0.75088 (15)	0.7214 (3)	−0.19493 (17)	0.0435 (6)	
C1	0.83949 (15)	0.3680 (3)	−0.04390 (15)	0.0334 (6)	
H1	0.8160	0.4564	−0.0300	0.040*	
C2	0.88960 (16)	0.3797 (4)	−0.09511 (16)	0.0408 (7)	
H2	0.9004	0.4747	−0.1144	0.049*	
C3	0.92279 (16)	0.2540 (4)	−0.11705 (16)	0.0408 (7)	
H3	0.9538	0.2594	−0.1546	0.049*	
C4	0.91060 (14)	0.1157 (4)	−0.08344 (15)	0.0349 (6)	
C5	0.94656 (15)	−0.0205 (4)	−0.09839 (17)	0.0413 (7)	
H5	0.9771	−0.0228	−0.1364	0.050*	
C6	0.93793 (16)	−0.1476 (4)	−0.05907 (18)	0.0429 (7)	
H6	0.9621	−0.2375	−0.0702	0.051*	
C7	0.89332 (15)	−0.1466 (3)	−0.00189 (17)	0.0364 (6)	
H7	0.8894	−0.2343	0.0270	0.044*	
C8	0.85549 (14)	−0.0183 (3)	0.01185 (15)	0.0286 (5)	
C9	0.86259 (13)	0.1132 (3)	−0.02987 (14)	0.0277 (5)	
C10	0.86537 (16)	−0.0119 (3)	0.17317 (16)	0.0370 (6)	
H10A	0.9035	0.0681	0.1772	0.044*	
H10B	0.8910	−0.1092	0.1753	0.044*	
H10C	0.8373	−0.0040	0.2150	0.044*	
C11	0.73838 (16)	−0.1577 (3)	0.07758 (19)	0.0391 (6)	
H11A	0.7694	−0.2480	0.0768	0.047*	
H11B	0.7002	−0.1523	0.0311	0.047*	
H11C	0.7126	−0.1611	0.1212	0.047*	
C12	0.66794 (15)	0.4179 (3)	−0.06857 (14)	0.0298 (5)	
H12	0.6936	0.3612	−0.1009	0.036*	
C13	0.62244 (16)	0.5391 (3)	−0.09962 (16)	0.0354 (6)	
H13	0.6166	0.5605	−0.1520	0.042*	
C14	0.58701 (15)	0.6251 (3)	−0.05472 (16)	0.0349 (6)	
H14	0.5590	0.7111	−0.0745	0.042*	
C15	0.59216 (14)	0.5853 (3)	0.02189 (15)	0.0287 (5)	
C16	0.55524 (15)	0.6643 (3)	0.07288 (17)	0.0343 (6)	
H16	0.5277	0.7531	0.0569	0.041*	
C17	0.55876 (15)	0.6142 (3)	0.14475 (17)	0.0357 (6)	
H17	0.5353	0.6702	0.1791	0.043*	
C18	0.59706 (15)	0.4793 (3)	0.16850 (16)	0.0320 (6)	
H18	0.5962	0.4416	0.2176	0.038*	
C19	0.63542 (14)	0.4026 (3)	0.12141 (14)	0.0262 (5)	
C20	0.63564 (14)	0.4568 (3)	0.04816 (14)	0.0250 (5)	
C21	0.60140 (15)	0.0941 (3)	0.13956 (17)	0.0348 (6)	
H21A	0.5720	0.0851	0.0882	0.042*	
H21B	0.5683	0.1325	0.1731	0.042*	
H21C	0.6212	−0.0044	0.1573	0.042*	
C22	0.73083 (16)	0.2268 (3)	0.23602 (14)	0.0332 (6)	
H22A	0.6946	0.2457	0.2698	0.040*	
H22B	0.7690	0.3069	0.2416	0.040*	
H22C	0.7563	0.1306	0.2489	0.040*	
C23	0.77162 (18)	0.8734 (3)	−0.21331 (18)	0.0415 (7)	
H23A	0.7415	0.9028	−0.2622	0.050*	
H23B	0.8261	0.8769	−0.2158	0.050*	
H23C	0.7611	0.9425	−0.1742	0.050*	

### (SP-4-2)-cis-Bis[8-(dimethylphosphanyl)quinoline-κ2N,P]nickel(II) bis(perchlorate) nitromethane monosolvate (1) Atomic displacement parameters (Å^2^)

**Table:**

	*U*^11^	*U*^22^	*U*^33^	*U*^12^	*U*^13^	*U*^23^
Ni1	0.02478 (17)	0.02146 (17)	0.02684 (18)	0.00232 (12)	0.00998 (12)	0.00229 (11)
Cl1	0.0286 (3)	0.0280 (3)	0.0311 (3)	0.0003 (2)	0.0078 (2)	−0.0003 (2)
Cl2	0.0402 (4)	0.0324 (3)	0.0390 (4)	−0.0014 (3)	0.0107 (3)	−0.0005 (3)
P1	0.0232 (3)	0.0227 (3)	0.0327 (3)	0.0020 (2)	0.0082 (2)	0.0015 (2)
P2	0.0240 (3)	0.0233 (3)	0.0246 (3)	0.0029 (2)	0.0078 (2)	0.0016 (2)
O1	0.0566 (13)	0.0350 (11)	0.0505 (13)	0.0162 (10)	0.0072 (11)	0.0061 (9)
O2	0.0594 (13)	0.0287 (10)	0.0360 (11)	−0.0060 (9)	0.0123 (10)	−0.0034 (8)
O3	0.0531 (13)	0.0633 (15)	0.0376 (12)	−0.0076 (11)	0.0227 (10)	−0.0053 (10)
O4	0.0267 (10)	0.0517 (13)	0.0637 (14)	−0.0025 (9)	0.0070 (9)	−0.0069 (11)
O5	0.0573 (18)	0.220 (5)	0.107 (3)	−0.033 (2)	0.0413 (19)	−0.094 (3)
O6	0.091 (2)	0.082 (2)	0.149 (3)	−0.041 (2)	0.059 (2)	−0.062 (2)
O7	0.164 (4)	0.120 (4)	0.159 (4)	−0.038 (3)	−0.041 (3)	0.091 (3)
O8	0.0430 (14)	0.095 (2)	0.080 (2)	0.0072 (14)	0.0111 (13)	−0.0257 (16)
O9	0.070 (2)	0.103 (3)	0.139 (3)	−0.0263 (18)	−0.029 (2)	0.086 (2)
O10	0.110 (2)	0.0643 (17)	0.0767 (19)	−0.0462 (18)	0.0471 (18)	−0.0385 (15)
N1	0.0243 (10)	0.0289 (10)	0.0280 (11)	−0.0045 (9)	0.0071 (8)	−0.0013 (8)
N2	0.0278 (10)	0.0213 (10)	0.0271 (10)	−0.0013 (8)	0.0074 (8)	0.0013 (8)
N3	0.0373 (13)	0.0317 (13)	0.0643 (18)	0.0005 (11)	0.0165 (13)	0.0012 (12)
C1	0.0315 (13)	0.0354 (14)	0.0337 (14)	−0.0072 (11)	0.0073 (11)	0.0006 (11)
C2	0.0332 (14)	0.0522 (18)	0.0378 (15)	−0.0148 (13)	0.0088 (12)	0.0072 (13)
C3	0.0261 (13)	0.066 (2)	0.0326 (14)	−0.0110 (14)	0.0113 (11)	−0.0016 (14)
C4	0.0211 (11)	0.0549 (18)	0.0286 (13)	−0.0042 (12)	0.0043 (10)	−0.0086 (12)
C5	0.0213 (12)	0.066 (2)	0.0377 (15)	0.0004 (13)	0.0072 (11)	−0.0178 (14)
C6	0.0263 (13)	0.0550 (19)	0.0467 (17)	0.0075 (13)	0.0051 (12)	−0.0227 (15)
C7	0.0275 (13)	0.0372 (15)	0.0432 (16)	0.0036 (12)	0.0026 (11)	−0.0096 (12)
C8	0.0204 (11)	0.0324 (13)	0.0328 (13)	−0.0007 (10)	0.0044 (10)	−0.0071 (10)
C9	0.0205 (11)	0.0343 (13)	0.0282 (12)	−0.0036 (10)	0.0038 (9)	−0.0080 (10)
C10	0.0324 (14)	0.0433 (16)	0.0354 (15)	0.0106 (12)	0.0069 (11)	0.0035 (12)
C11	0.0338 (14)	0.0282 (13)	0.0570 (18)	−0.0022 (12)	0.0128 (13)	0.0031 (13)
C12	0.0320 (13)	0.0285 (12)	0.0295 (13)	−0.0043 (11)	0.0068 (10)	0.0019 (10)
C13	0.0365 (14)	0.0350 (14)	0.0332 (14)	−0.0058 (12)	0.0023 (11)	0.0129 (11)
C14	0.0321 (13)	0.0256 (13)	0.0450 (16)	−0.0024 (11)	0.0014 (12)	0.0106 (11)
C15	0.0244 (11)	0.0212 (11)	0.0394 (14)	−0.0027 (10)	0.0029 (10)	0.0006 (10)
C16	0.0273 (12)	0.0226 (12)	0.0517 (17)	0.0033 (10)	0.0035 (12)	−0.0029 (11)
C17	0.0292 (13)	0.0324 (14)	0.0457 (16)	0.0073 (11)	0.0075 (11)	−0.0120 (12)
C18	0.0288 (12)	0.0345 (14)	0.0330 (14)	0.0043 (11)	0.0065 (10)	−0.0053 (11)
C19	0.0247 (11)	0.0256 (12)	0.0279 (12)	0.0022 (10)	0.0039 (9)	−0.0016 (9)
C20	0.0239 (11)	0.0199 (11)	0.0309 (12)	−0.0014 (9)	0.0043 (9)	−0.0015 (9)
C21	0.0294 (13)	0.0337 (14)	0.0436 (15)	−0.0020 (11)	0.0128 (11)	0.0019 (12)
C22	0.0383 (14)	0.0352 (14)	0.0256 (13)	0.0099 (12)	0.0047 (11)	0.0011 (10)
C23	0.0407 (15)	0.0336 (15)	0.0501 (17)	−0.0029 (13)	0.0081 (13)	0.0051 (13)

### (SP-4-2)-cis-Bis[8-(dimethylphosphanyl)quinoline-κ2N,P]nickel(II) bis(perchlorate) nitromethane monosolvate (1) Geometric parameters (Å, º)

**Table:**

Ni1—N1	1.970 (2)	C6—C7	1.412 (4)
Ni1—N2	1.982 (2)	C6—H6	0.9500
Ni1—P2	2.1534 (7)	C7—C8	1.376 (4)
Ni1—P1	2.1576 (7)	C7—H7	0.9500
Cl1—O3	1.428 (2)	C8—C9	1.413 (4)
Cl1—O2	1.433 (2)	C10—H10A	0.9800
Cl1—O1	1.438 (2)	C10—H10B	0.9800
Cl1—O4	1.438 (2)	C10—H10C	0.9800
Cl2—O7	1.376 (4)	C11—H11A	0.9800
Cl2—O5	1.381 (3)	C11—H11B	0.9800
Cl2—O6	1.408 (3)	C11—H11C	0.9800
Cl2—O8	1.446 (3)	C12—C13	1.406 (4)
P1—C8	1.800 (3)	C12—H12	0.9500
P1—C11	1.809 (3)	C13—C14	1.353 (4)
P1—C10	1.810 (3)	C13—H13	0.9500
P2—C22	1.807 (3)	C14—C15	1.413 (4)
P2—C21	1.807 (3)	C14—H14	0.9500
P2—C19	1.808 (3)	C15—C16	1.413 (4)
O9—N3	1.199 (4)	C15—C20	1.417 (3)
O10—N3	1.209 (4)	C16—C17	1.361 (4)
N1—C1	1.327 (3)	C16—H16	0.9500
N1—C9	1.381 (3)	C17—C18	1.413 (4)
N2—C12	1.322 (3)	C17—H17	0.9500
N2—C20	1.378 (3)	C18—C19	1.367 (3)
N3—C23	1.462 (4)	C18—H18	0.9500
C1—C2	1.400 (4)	C19—C20	1.407 (3)
C1—H1	0.9500	C21—H21A	0.9800
C2—C3	1.362 (5)	C21—H21B	0.9800
C2—H2	0.9500	C21—H21C	0.9800
C3—C4	1.411 (4)	C22—H22A	0.9800
C3—H3	0.9500	C22—H22B	0.9800
C4—C9	1.402 (3)	C22—H22C	0.9800
C4—C5	1.424 (4)	C23—H23A	0.9800
C5—C6	1.363 (5)	C23—H23B	0.9800
C5—H5	0.9500	C23—H23C	0.9800
			
N1—Ni1—N2	97.01 (9)	C9—C8—P1	113.84 (18)
N1—Ni1—P2	166.27 (7)	N1—C9—C4	121.7 (2)
N2—Ni1—P2	85.97 (6)	N1—C9—C8	117.9 (2)
N1—Ni1—P1	86.13 (7)	C4—C9—C8	120.3 (2)
N2—Ni1—P1	165.68 (6)	P1—C10—H10A	109.5
P2—Ni1—P1	94.29 (3)	P1—C10—H10B	109.5
O3—Cl1—O2	110.15 (13)	H10A—C10—H10B	109.5
O3—Cl1—O1	109.95 (14)	P1—C10—H10C	109.5
O2—Cl1—O1	109.60 (13)	H10A—C10—H10C	109.5
O3—Cl1—O4	109.09 (14)	H10B—C10—H10C	109.5
O2—Cl1—O4	109.61 (13)	P1—C11—H11A	109.5
O1—Cl1—O4	108.41 (14)	P1—C11—H11B	109.5
O7—Cl2—O5	111.9 (4)	H11A—C11—H11B	109.5
O7—Cl2—O6	106.6 (3)	P1—C11—H11C	109.5
O5—Cl2—O6	111.0 (2)	H11A—C11—H11C	109.5
O7—Cl2—O8	105.4 (3)	H11B—C11—H11C	109.5
O5—Cl2—O8	110.3 (2)	N2—C12—C13	123.4 (2)
O6—Cl2—O8	111.5 (2)	N2—C12—H12	118.3
C8—P1—C11	105.12 (13)	C13—C12—H12	118.3
C8—P1—C10	105.93 (12)	C14—C13—C12	119.8 (3)
C11—P1—C10	106.05 (15)	C14—C13—H13	120.1
C8—P1—Ni1	99.90 (9)	C12—C13—H13	120.1
C11—P1—Ni1	117.20 (10)	C13—C14—C15	119.2 (2)
C10—P1—Ni1	120.75 (10)	C13—C14—H14	120.4
C22—P2—C21	105.52 (14)	C15—C14—H14	120.4
C22—P2—C19	106.08 (12)	C14—C15—C16	123.7 (2)
C21—P2—C19	104.32 (12)	C14—C15—C20	117.7 (2)
C22—P2—Ni1	118.50 (10)	C16—C15—C20	118.6 (2)
C21—P2—Ni1	120.92 (10)	C17—C16—C15	120.6 (2)
C19—P2—Ni1	99.35 (8)	C17—C16—H16	119.7
C1—N1—C9	117.8 (2)	C15—C16—H16	119.7
C1—N1—Ni1	123.61 (18)	C16—C17—C18	120.3 (2)
C9—N1—Ni1	118.53 (17)	C16—C17—H17	119.8
C12—N2—C20	117.4 (2)	C18—C17—H17	119.8
C12—N2—Ni1	124.45 (18)	C19—C18—C17	120.6 (3)
C20—N2—Ni1	118.18 (16)	C19—C18—H18	119.7
O9—N3—O10	123.2 (3)	C17—C18—H18	119.7
O9—N3—C23	118.2 (3)	C18—C19—C20	119.8 (2)
O10—N3—C23	118.6 (3)	C18—C19—P2	126.2 (2)
N1—C1—C2	123.0 (3)	C20—C19—P2	113.68 (18)
N1—C1—H1	118.5	N2—C20—C19	118.0 (2)
C2—C1—H1	118.5	N2—C20—C15	122.1 (2)
C3—C2—C1	119.5 (3)	C19—C20—C15	119.8 (2)
C3—C2—H2	120.3	P2—C21—H21A	109.5
C1—C2—H2	120.3	P2—C21—H21B	109.5
C2—C3—C4	119.3 (2)	H21A—C21—H21B	109.5
C2—C3—H3	120.3	P2—C21—H21C	109.5
C4—C3—H3	120.3	H21A—C21—H21C	109.5
C9—C4—C3	118.1 (3)	H21B—C21—H21C	109.5
C9—C4—C5	117.9 (3)	P2—C22—H22A	109.5
C3—C4—C5	123.9 (3)	P2—C22—H22B	109.5
C6—C5—C4	121.1 (3)	H22A—C22—H22B	109.5
C6—C5—H5	119.4	P2—C22—H22C	109.5
C4—C5—H5	119.4	H22A—C22—H22C	109.5
C5—C6—C7	120.4 (3)	H22B—C22—H22C	109.5
C5—C6—H6	119.8	N3—C23—H23A	109.5
C7—C6—H6	119.8	N3—C23—H23B	109.5
C8—C7—C6	119.7 (3)	H23A—C23—H23B	109.5
C8—C7—H7	120.1	N3—C23—H23C	109.5
C6—C7—H7	120.1	H23A—C23—H23C	109.5
C7—C8—C9	120.2 (2)	H23B—C23—H23C	109.5
C7—C8—P1	125.8 (2)		
			
C9—N1—C1—C2	5.1 (4)	C20—N2—C12—C13	3.7 (4)
Ni1—N1—C1—C2	−173.7 (2)	Ni1—N2—C12—C13	−175.97 (19)
N1—C1—C2—C3	1.3 (4)	N2—C12—C13—C14	2.1 (4)
C1—C2—C3—C4	−4.8 (4)	C12—C13—C14—C15	−4.3 (4)
C2—C3—C4—C9	1.8 (4)	C13—C14—C15—C16	−177.8 (3)
C2—C3—C4—C5	−175.6 (3)	C13—C14—C15—C20	0.9 (4)
C9—C4—C5—C6	−3.4 (4)	C14—C15—C16—C17	175.8 (3)
C3—C4—C5—C6	173.9 (3)	C20—C15—C16—C17	−2.8 (4)
C4—C5—C6—C7	−0.5 (4)	C15—C16—C17—C18	−2.4 (4)
C5—C6—C7—C8	3.0 (4)	C16—C17—C18—C19	4.4 (4)
C6—C7—C8—C9	−1.5 (4)	C17—C18—C19—C20	−1.2 (4)
C6—C7—C8—P1	−177.4 (2)	C17—C18—C19—P2	−174.3 (2)
C11—P1—C8—C7	−47.4 (3)	C22—P2—C19—C18	−44.2 (3)
C10—P1—C8—C7	64.6 (3)	C21—P2—C19—C18	67.0 (3)
Ni1—P1—C8—C7	−169.2 (2)	Ni1—P2—C19—C18	−167.6 (2)
C11—P1—C8—C9	136.4 (2)	C22—P2—C19—C20	142.27 (19)
C10—P1—C8—C9	−111.6 (2)	C21—P2—C19—C20	−106.6 (2)
Ni1—P1—C8—C9	14.54 (19)	Ni1—P2—C19—C20	18.86 (19)
C1—N1—C9—C4	−8.2 (4)	C12—N2—C20—C19	170.6 (2)
Ni1—N1—C9—C4	170.70 (18)	Ni1—N2—C20—C19	−9.7 (3)
C1—N1—C9—C8	169.3 (2)	C12—N2—C20—C15	−7.3 (3)
Ni1—N1—C9—C8	−11.8 (3)	Ni1—N2—C20—C15	172.42 (18)
C3—C4—C9—N1	4.8 (4)	C18—C19—C20—N2	178.1 (2)
C5—C4—C9—N1	−177.7 (2)	P2—C19—C20—N2	−8.0 (3)
C3—C4—C9—C8	−172.6 (2)	C18—C19—C20—C15	−4.0 (4)
C5—C4—C9—C8	4.9 (4)	P2—C19—C20—C15	169.96 (18)
C7—C8—C9—N1	179.9 (2)	C14—C15—C20—N2	5.1 (4)
P1—C8—C9—N1	−3.6 (3)	C16—C15—C20—N2	−176.2 (2)
C7—C8—C9—C4	−2.6 (4)	C14—C15—C20—C19	−172.7 (2)
P1—C8—C9—C4	173.89 (19)	C16—C15—C20—C19	6.0 (4)

### (SP-4-2)-cis-Bis[8-(dimethylphosphanyl)quinoline-κ2N,P]platinum(II) bis(tetrafluoroborate) acetonitrile monosolvate (2) Crystal data

**Table:**

[Pt(C_11_H_12_NP)_2_](BF_4_)_2_·C_2_H_3_N	*F*(000) = 1528
*M**_r_* = 788.13	*D*_x_ = 1.882 Mg m^−^^3^
Monoclinic, *P*2_1_/*n*	Mo *K*α radiation, λ = 0.71075 Å
*a* = 7.9102 (3) Å	Cell parameters from 31045 reflections
*b* = 21.0833 (5) Å	θ = 3.1–27.5°
*c* = 16.7519 (4) Å	µ = 5.23 mm^−^^1^
β = 95.3931 (11)°	*T* = 188 K
*V* = 2781.42 (15) Å^3^	Platelet, colorless
*Z* = 4	0.30 × 0.30 × 0.10 mm

### (SP-4-2)-cis-Bis[8-(dimethylphosphanyl)quinoline-κ2N,P]platinum(II) bis(tetrafluoroborate) acetonitrile monosolvate (2) Data collection

**Table:**

Rigaku R-AXIS RAPID diffractometer	6350 independent reflections
Radiation source: fine-focus sealed tube	5435 reflections with *I* > 2σ(*I*)
Detector resolution: 10.000 pixels mm^-1^	*R*_int_ = 0.042
ω scans	θ_max_ = 27.5°, θ_min_ = 3.1°
Absorption correction: numerical (NUMABS; Rigaku, 1999)	*h* = −10→10
*T*_min_ = 0.338, *T*_max_ = 0.594	*k* = −27→27
43967 measured reflections	*l* = −21→21

### (SP-4-2)-cis-Bis[8-(dimethylphosphanyl)quinoline-κ2N,P]platinum(II) bis(tetrafluoroborate) acetonitrile monosolvate (2) Refinement

**Table:**

Refinement on *F*^2^	0 restraints
Least-squares matrix: full	Hydrogen site location: inferred from neighbouring sites
*R*[*F*^2^ > 2σ(*F*^2^)] = 0.033	H-atom parameters constrained
*wR*(*F*^2^) = 0.084	*w* = 1/[σ^2^(*F*_o_^2^) + (0.0382*P*)^2^ + 9.8949*P*] where *P* = (*F*_o_^2^ + 2*F*_c_^2^)/3
*S* = 1.07	(Δ/σ)_max_ = 0.001
6350 reflections	Δρ_max_ = 1.42 e Å^−^^3^
366 parameters	Δρ_min_ = −1.44 e Å^−^^3^

### (SP-4-2)-cis-Bis[8-(dimethylphosphanyl)quinoline-κ2N,P]platinum(II) bis(tetrafluoroborate) acetonitrile monosolvate (2) Special details

**Table:**

Geometry. All esds (except the esd in the dihedral angle between two l.s. planes) are estimated using the full covariance matrix. The cell esds are taken into account individually in the estimation of esds in distances, angles and torsion angles; correlations between esds in cell parameters are only used when they are defined by crystal symmetry. An approximate (isotropic) treatment of cell esds is used for estimating esds involving l.s. planes.

### (SP-4-2)-cis-Bis[8-(dimethylphosphanyl)quinoline-κ2N,P]platinum(II) bis(tetrafluoroborate) acetonitrile monosolvate (2) Fractional atomic coordinates and isotropic or equivalent isotropic displacement parameters (Å^2^)

**Table:**

	*x*	*y*	*z*	*U*_iso_*/*U*_eq_	Occ. (<1)
Pt1	0.64274 (2)	0.17364 (2)	0.20553 (2)	0.02183 (7)	
P1	0.50347 (15)	0.24824 (5)	0.26806 (7)	0.0237 (2)	
P2	0.80277 (15)	0.23745 (6)	0.13781 (7)	0.0247 (2)	
F1	1.2369 (6)	−0.1256 (2)	0.0823 (5)	0.136 (3)	
F2A	1.5089 (15)	−0.1511 (5)	0.0808 (7)	0.089 (2)	0.573 (10)
F3A	1.4191 (13)	−0.0455 (4)	0.0691 (7)	0.089 (2)	0.573 (10)
F4A	1.3835 (12)	−0.1067 (4)	0.1779 (6)	0.089 (2)	0.573 (10)
F2B	1.4392 (16)	−0.1049 (8)	0.0213 (10)	0.121 (4)	0.427 (10)
F3B	1.4100 (18)	−0.0423 (9)	0.1216 (12)	0.121 (4)	0.427 (10)
F4B	1.520 (2)	−0.1430 (9)	0.1262 (12)	0.121 (4)	0.427 (10)
F5	0.0397 (6)	0.3460 (3)	−0.0167 (5)	0.115 (2)	
F6	0.2266 (7)	0.4071 (2)	−0.0644 (3)	0.0883 (15)	
F7	0.1968 (10)	0.4023 (3)	0.0675 (3)	0.126 (3)	
F8	0.3139 (5)	0.31996 (17)	0.0067 (2)	0.0566 (10)	
N1	0.4883 (5)	0.11161 (18)	0.2672 (2)	0.0256 (8)	
N2	0.7738 (5)	0.10240 (18)	0.1453 (2)	0.0270 (8)	
N3	0.8437 (7)	−0.0443 (3)	0.3316 (4)	0.0521 (13)	
C1	0.5191 (7)	0.0503 (2)	0.2795 (3)	0.0342 (11)	
H1	0.6285	0.0343	0.2714	0.041*	
C2	0.3965 (8)	0.0078 (3)	0.3041 (3)	0.0411 (13)	
H2	0.4219	−0.0361	0.3103	0.049*	
C3	0.2415 (7)	0.0304 (3)	0.3190 (3)	0.0395 (12)	
H3	0.1547	0.0020	0.3317	0.047*	
C4	0.2103 (6)	0.0963 (2)	0.3155 (3)	0.0310 (10)	
C5	0.0599 (6)	0.1251 (3)	0.3391 (3)	0.0350 (11)	
H5	−0.0327	0.0992	0.3512	0.042*	
C6	0.0477 (7)	0.1893 (3)	0.3444 (3)	0.0346 (11)	
H6	−0.0519	0.2077	0.3620	0.042*	
C7	0.1812 (6)	0.2289 (2)	0.3239 (3)	0.0307 (10)	
H7	0.1727	0.2736	0.3298	0.037*	
C8	0.3228 (6)	0.2031 (2)	0.2955 (3)	0.0253 (9)	
C9	0.3391 (6)	0.1368 (2)	0.2909 (3)	0.0254 (9)	
C10	0.6062 (6)	0.2807 (2)	0.3599 (3)	0.0328 (11)	
H10A	0.6548	0.2461	0.3938	0.039*	
H10B	0.6968	0.3097	0.3474	0.039*	
H10C	0.5227	0.3038	0.3884	0.039*	
C11	0.4207 (7)	0.3163 (2)	0.2120 (3)	0.0353 (11)	
H11A	0.3625	0.3023	0.1609	0.042*	
H11B	0.3403	0.3389	0.2429	0.042*	
H11C	0.5144	0.3447	0.2019	0.042*	
C12	0.7232 (7)	0.0429 (2)	0.1333 (3)	0.0333 (11)	
H12	0.6088	0.0325	0.1405	0.040*	
C13	0.8316 (7)	−0.0059 (2)	0.1104 (3)	0.0380 (12)	
H13	0.7911	−0.0482	0.1044	0.046*	
C14	0.9946 (7)	0.0083 (3)	0.0970 (3)	0.0392 (12)	
H14	1.0717	−0.0244	0.0861	0.047*	
C15	1.0478 (6)	0.0725 (2)	0.0996 (3)	0.0311 (10)	
C16	1.2074 (6)	0.0932 (3)	0.0757 (3)	0.0392 (12)	
H16	1.2897	0.0628	0.0632	0.047*	
C17	1.2421 (7)	0.1564 (3)	0.0707 (3)	0.0409 (13)	
H17	1.3472	0.1696	0.0530	0.049*	
C18	1.1258 (6)	0.2021 (3)	0.0913 (3)	0.0350 (11)	
H18	1.1518	0.2459	0.0865	0.042*	
C19	0.9729 (6)	0.1843 (2)	0.1186 (3)	0.0268 (10)	
C20	0.9326 (6)	0.1191 (2)	0.1230 (3)	0.0256 (9)	
C21	0.8949 (7)	0.3084 (2)	0.1834 (3)	0.0349 (11)	
H21A	0.8043	0.3379	0.1944	0.042*	
H21B	0.9617	0.2974	0.2338	0.042*	
H21C	0.9688	0.3284	0.1469	0.042*	
C22	0.7056 (7)	0.2620 (3)	0.0406 (3)	0.0354 (11)	
H22A	0.6527	0.2252	0.0124	0.042*	
H22B	0.6189	0.2942	0.0476	0.042*	
H22C	0.7926	0.2798	0.0091	0.042*	
C23	0.8162 (8)	−0.0480 (3)	0.3952 (4)	0.0437 (13)	
C24	0.7834 (13)	−0.0553 (4)	0.4779 (4)	0.078 (2)	
H24A	0.7717	−0.0134	0.5021	0.094*	
H24B	0.8781	−0.0780	0.5071	0.094*	
H24C	0.6783	−0.0794	0.4808	0.094*	
B1	1.3953 (8)	−0.1035 (3)	0.0979 (4)	0.0361 (13)	
B2	0.2039 (8)	0.3716 (3)	0.0001 (4)	0.0370 (13)	

### (SP-4-2)-cis-Bis[8-(dimethylphosphanyl)quinoline-κ2N,P]platinum(II) bis(tetrafluoroborate) acetonitrile monosolvate (2) Atomic displacement parameters (Å^2^)

**Table:**

	*U*^11^	*U*^22^	*U*^33^	*U*^12^	*U*^13^	*U*^23^
Pt1	0.02079 (9)	0.02222 (10)	0.02284 (10)	0.00020 (6)	0.00396 (6)	0.00096 (7)
P1	0.0234 (6)	0.0233 (5)	0.0243 (6)	0.0018 (4)	0.0025 (4)	0.0014 (5)
P2	0.0221 (6)	0.0261 (6)	0.0258 (6)	−0.0021 (4)	0.0023 (4)	0.0032 (5)
F1	0.040 (2)	0.051 (3)	0.317 (10)	−0.003 (2)	0.017 (4)	0.009 (4)
F2A	0.104 (4)	0.057 (3)	0.106 (5)	0.009 (3)	0.014 (4)	0.015 (3)
F3A	0.104 (4)	0.057 (3)	0.106 (5)	0.009 (3)	0.014 (4)	0.015 (3)
F4A	0.104 (4)	0.057 (3)	0.106 (5)	0.009 (3)	0.014 (4)	0.015 (3)
F2B	0.071 (5)	0.136 (8)	0.158 (10)	−0.024 (5)	0.031 (6)	−0.015 (7)
F3B	0.071 (5)	0.136 (8)	0.158 (10)	−0.024 (5)	0.031 (6)	−0.015 (7)
F4B	0.071 (5)	0.136 (8)	0.158 (10)	−0.024 (5)	0.031 (6)	−0.015 (7)
F5	0.052 (3)	0.081 (3)	0.215 (7)	0.001 (2)	0.032 (4)	0.012 (4)
F6	0.108 (4)	0.087 (3)	0.075 (3)	0.032 (3)	0.037 (3)	0.040 (3)
F7	0.207 (7)	0.110 (4)	0.057 (3)	0.077 (5)	−0.008 (4)	−0.027 (3)
F8	0.053 (2)	0.054 (2)	0.062 (2)	0.0172 (17)	0.0060 (18)	0.0040 (18)
N1	0.0241 (19)	0.0264 (19)	0.0269 (19)	0.0009 (15)	0.0066 (15)	0.0044 (16)
N2	0.028 (2)	0.027 (2)	0.0252 (19)	−0.0004 (16)	0.0028 (16)	−0.0020 (16)
N3	0.052 (3)	0.049 (3)	0.057 (3)	0.003 (2)	0.013 (3)	0.004 (3)
C1	0.040 (3)	0.028 (2)	0.037 (3)	0.004 (2)	0.012 (2)	0.004 (2)
C2	0.058 (4)	0.026 (3)	0.042 (3)	0.000 (2)	0.018 (3)	0.006 (2)
C3	0.045 (3)	0.034 (3)	0.042 (3)	−0.010 (2)	0.014 (2)	0.003 (2)
C4	0.030 (2)	0.037 (3)	0.026 (2)	−0.005 (2)	0.0045 (19)	0.000 (2)
C5	0.025 (2)	0.049 (3)	0.032 (3)	−0.003 (2)	0.007 (2)	0.004 (2)
C6	0.030 (3)	0.047 (3)	0.028 (2)	0.009 (2)	0.006 (2)	0.005 (2)
C7	0.030 (2)	0.038 (3)	0.025 (2)	0.006 (2)	0.0046 (19)	0.001 (2)
C8	0.026 (2)	0.031 (2)	0.019 (2)	0.0008 (18)	0.0018 (17)	0.0029 (18)
C9	0.026 (2)	0.029 (2)	0.022 (2)	0.0007 (18)	0.0033 (18)	0.0014 (18)
C10	0.032 (3)	0.036 (3)	0.030 (2)	0.000 (2)	0.001 (2)	−0.007 (2)
C11	0.033 (3)	0.031 (3)	0.043 (3)	0.004 (2)	0.006 (2)	0.009 (2)
C12	0.035 (3)	0.030 (2)	0.036 (3)	−0.004 (2)	0.010 (2)	−0.004 (2)
C13	0.052 (3)	0.025 (2)	0.038 (3)	0.000 (2)	0.013 (2)	−0.004 (2)
C14	0.047 (3)	0.038 (3)	0.033 (3)	0.013 (2)	0.007 (2)	−0.002 (2)
C15	0.029 (2)	0.039 (3)	0.025 (2)	0.006 (2)	0.0046 (19)	−0.003 (2)
C16	0.026 (3)	0.057 (3)	0.034 (3)	0.009 (2)	0.004 (2)	−0.004 (3)
C17	0.026 (3)	0.062 (4)	0.035 (3)	−0.007 (2)	0.007 (2)	−0.010 (3)
C18	0.028 (2)	0.045 (3)	0.033 (3)	−0.007 (2)	0.008 (2)	−0.003 (2)
C19	0.024 (2)	0.033 (2)	0.024 (2)	−0.0016 (18)	0.0028 (18)	−0.0035 (19)
C20	0.022 (2)	0.031 (2)	0.023 (2)	0.0034 (18)	0.0017 (17)	0.0004 (19)
C21	0.030 (3)	0.029 (2)	0.046 (3)	−0.004 (2)	0.001 (2)	−0.002 (2)
C22	0.034 (3)	0.043 (3)	0.029 (2)	−0.001 (2)	0.000 (2)	0.007 (2)
C23	0.044 (3)	0.030 (3)	0.057 (4)	−0.006 (2)	0.005 (3)	−0.001 (3)
C24	0.125 (7)	0.057 (4)	0.054 (4)	−0.025 (5)	0.020 (5)	0.005 (4)
B1	0.038 (3)	0.028 (3)	0.044 (4)	0.003 (2)	0.010 (3)	−0.002 (3)
B2	0.043 (3)	0.034 (3)	0.036 (3)	0.005 (3)	0.014 (3)	0.001 (3)

### (SP-4-2)-cis-Bis[8-(dimethylphosphanyl)quinoline-κ2N,P]platinum(II) bis(tetrafluoroborate) acetonitrile monosolvate (2) Geometric parameters (Å, º)

**Table:**

Pt1—N1	2.123 (4)	C6—C7	1.414 (7)
Pt1—N2	2.132 (4)	C6—H6	0.9500
Pt1—P2	2.2293 (12)	C7—C8	1.371 (6)
Pt1—P1	2.2365 (12)	C7—H7	0.9500
P1—C11	1.804 (5)	C8—C9	1.405 (7)
P1—C10	1.805 (5)	C10—H10A	0.9800
P1—C8	1.812 (5)	C10—H10B	0.9800
P2—C21	1.802 (5)	C10—H10C	0.9800
P2—C19	1.804 (5)	C11—H11A	0.9800
P2—C22	1.810 (5)	C11—H11B	0.9800
F1—B1	1.340 (8)	C11—H11C	0.9800
F2A—B1	1.395 (13)	C12—C13	1.416 (7)
F3A—B1	1.334 (11)	C12—H12	0.9500
F4A—B1	1.353 (11)	C13—C14	1.363 (8)
F2B—B1	1.361 (17)	C13—H13	0.9500
F3B—B1	1.351 (18)	C14—C15	1.416 (8)
F4B—B1	1.35 (2)	C14—H14	0.9500
F5—B2	1.410 (8)	C15—C20	1.421 (6)
F6—B2	1.341 (7)	C15—C16	1.428 (7)
F7—B2	1.309 (8)	C16—C17	1.366 (8)
F8—B2	1.391 (7)	C16—H16	0.9500
N1—C1	1.329 (6)	C17—C18	1.398 (8)
N1—C9	1.386 (6)	C17—H17	0.9500
N2—C12	1.326 (6)	C18—C19	1.384 (7)
N2—C20	1.389 (6)	C18—H18	0.9500
N3—C23	1.110 (8)	C19—C20	1.414 (6)
C1—C2	1.409 (7)	C21—H21A	0.9800
C1—H1	0.9500	C21—H21B	0.9800
C2—C3	1.360 (8)	C21—H21C	0.9800
C2—H2	0.9500	C22—H22A	0.9800
C3—C4	1.410 (7)	C22—H22B	0.9800
C3—H3	0.9500	C22—H22C	0.9800
C4—C9	1.421 (7)	C23—C24	1.441 (9)
C4—C5	1.424 (7)	C24—H24A	0.9800
C5—C6	1.361 (8)	C24—H24B	0.9800
C5—H5	0.9500	C24—H24C	0.9800
			
N1—Pt1—N2	97.13 (15)	N2—C12—H12	118.4
N1—Pt1—P2	178.51 (11)	C13—C12—H12	118.4
N2—Pt1—P2	81.93 (11)	C14—C13—C12	119.4 (5)
N1—Pt1—P1	82.76 (11)	C14—C13—H13	120.3
N2—Pt1—P1	179.55 (11)	C12—C13—H13	120.3
P2—Pt1—P1	98.17 (4)	C13—C14—C15	119.2 (5)
C11—P1—C10	104.7 (3)	C13—C14—H14	120.4
C11—P1—C8	107.1 (2)	C15—C14—H14	120.4
C10—P1—C8	106.6 (2)	C14—C15—C20	118.3 (5)
C11—P1—Pt1	119.12 (19)	C14—C15—C16	123.4 (5)
C10—P1—Pt1	117.60 (17)	C20—C15—C16	118.2 (5)
C8—P1—Pt1	100.60 (16)	C17—C16—C15	120.2 (5)
C21—P2—C19	108.2 (2)	C17—C16—H16	119.9
C21—P2—C22	105.4 (3)	C15—C16—H16	119.9
C19—P2—C22	106.1 (2)	C16—C17—C18	121.2 (5)
C21—P2—Pt1	120.76 (19)	C16—C17—H17	119.4
C19—P2—Pt1	100.65 (16)	C18—C17—H17	119.4
C22—P2—Pt1	114.64 (18)	C19—C18—C17	120.6 (5)
C1—N1—C9	118.5 (4)	C19—C18—H18	119.7
C1—N1—Pt1	124.7 (3)	C17—C18—H18	119.7
C9—N1—Pt1	116.6 (3)	C18—C19—C20	119.3 (4)
C12—N2—C20	117.8 (4)	C18—C19—P2	125.4 (4)
C12—N2—Pt1	125.8 (3)	C20—C19—P2	114.7 (3)
C20—N2—Pt1	116.2 (3)	N2—C20—C19	118.3 (4)
N1—C1—C2	122.9 (5)	N2—C20—C15	121.2 (4)
N1—C1—H1	118.6	C19—C20—C15	120.3 (4)
C2—C1—H1	118.6	P2—C21—H21A	109.5
C3—C2—C1	119.2 (5)	P2—C21—H21B	109.5
C3—C2—H2	120.4	H21A—C21—H21B	109.5
C1—C2—H2	120.4	P2—C21—H21C	109.5
C2—C3—C4	119.7 (5)	H21A—C21—H21C	109.5
C2—C3—H3	120.2	H21B—C21—H21C	109.5
C4—C3—H3	120.2	P2—C22—H22A	109.5
C3—C4—C9	118.5 (5)	P2—C22—H22B	109.5
C3—C4—C5	123.8 (5)	H22A—C22—H22B	109.5
C9—C4—C5	117.6 (5)	P2—C22—H22C	109.5
C6—C5—C4	120.6 (5)	H22A—C22—H22C	109.5
C6—C5—H5	119.7	H22B—C22—H22C	109.5
C4—C5—H5	119.7	N3—C23—C24	177.7 (7)
C5—C6—C7	120.8 (5)	C23—C24—H24A	109.5
C5—C6—H6	119.6	C23—C24—H24B	109.5
C7—C6—H6	119.6	H24A—C24—H24B	109.5
C8—C7—C6	120.2 (5)	C23—C24—H24C	109.5
C8—C7—H7	119.9	H24A—C24—H24C	109.5
C6—C7—H7	119.9	H24B—C24—H24C	109.5
C7—C8—C9	119.8 (4)	F3A—B1—F1	114.2 (7)
C7—C8—P1	124.7 (4)	F1—B1—F4B	119.8 (9)
C9—C8—P1	115.5 (3)	F1—B1—F3B	116.3 (8)
N1—C9—C8	118.8 (4)	F4B—B1—F3B	116.6 (12)
N1—C9—C4	120.3 (4)	F3A—B1—F4A	115.4 (8)
C8—C9—C4	120.7 (4)	F1—B1—F4A	91.4 (7)
P1—C10—H10A	109.5	F1—B1—F2B	97.2 (8)
P1—C10—H10B	109.5	F4B—B1—F2B	94.3 (11)
H10A—C10—H10B	109.5	F3B—B1—F2B	106.0 (11)
P1—C10—H10C	109.5	F3A—B1—F2A	118.0 (8)
H10A—C10—H10C	109.5	F1—B1—F2A	108.5 (7)
H10B—C10—H10C	109.5	F4A—B1—F2A	105.8 (7)
P1—C11—H11A	109.5	F7—B2—F6	116.0 (6)
P1—C11—H11B	109.5	F7—B2—F8	113.3 (6)
H11A—C11—H11B	109.5	F6—B2—F8	111.8 (5)
P1—C11—H11C	109.5	F7—B2—F5	104.4 (6)
H11A—C11—H11C	109.5	F6—B2—F5	104.1 (6)
H11B—C11—H11C	109.5	F8—B2—F5	105.9 (5)
N2—C12—C13	123.2 (5)		
			
C9—N1—C1—C2	10.0 (7)	C20—N2—C12—C13	9.6 (7)
Pt1—N1—C1—C2	−165.3 (4)	Pt1—N2—C12—C13	−164.6 (4)
N1—C1—C2—C3	−2.5 (8)	N2—C12—C13—C14	−2.3 (8)
C1—C2—C3—C4	−4.8 (8)	C12—C13—C14—C15	−5.4 (8)
C2—C3—C4—C9	4.3 (8)	C13—C14—C15—C20	5.4 (7)
C2—C3—C4—C5	−172.4 (5)	C13—C14—C15—C16	−171.1 (5)
C3—C4—C5—C6	171.1 (5)	C14—C15—C16—C17	172.3 (5)
C9—C4—C5—C6	−5.6 (7)	C20—C15—C16—C17	−4.2 (7)
C4—C5—C6—C7	2.3 (8)	C15—C16—C17—C18	2.2 (8)
C5—C6—C7—C8	2.4 (7)	C16—C17—C18—C19	1.1 (8)
C6—C7—C8—C9	−3.5 (7)	C17—C18—C19—C20	−2.1 (7)
C6—C7—C8—P1	−179.3 (4)	C17—C18—C19—P2	−173.6 (4)
C11—P1—C8—C7	−43.4 (5)	C21—P2—C19—C18	−39.4 (5)
C10—P1—C8—C7	68.3 (4)	C22—P2—C19—C18	73.3 (5)
Pt1—P1—C8—C7	−168.5 (4)	Pt1—P2—C19—C18	−167.0 (4)
C11—P1—C8—C9	140.6 (4)	C21—P2—C19—C20	148.8 (4)
C10—P1—C8—C9	−107.7 (4)	C22—P2—C19—C20	−98.5 (4)
Pt1—P1—C8—C9	15.5 (3)	Pt1—P2—C19—C20	21.2 (4)
C1—N1—C9—C8	165.5 (4)	C12—N2—C20—C19	166.2 (4)
Pt1—N1—C9—C8	−18.9 (5)	Pt1—N2—C20—C19	−19.0 (5)
C1—N1—C9—C4	−10.1 (7)	C12—N2—C20—C15	−9.3 (7)
Pt1—N1—C9—C4	165.5 (3)	Pt1—N2—C20—C15	165.4 (3)
C7—C8—C9—N1	−175.6 (4)	C18—C19—C20—N2	−175.7 (4)
P1—C8—C9—N1	0.6 (5)	P2—C19—C20—N2	−3.3 (6)
C7—C8—C9—C4	−0.1 (7)	C18—C19—C20—C15	−0.1 (7)
P1—C8—C9—C4	176.2 (3)	P2—C19—C20—C15	172.3 (4)
C3—C4—C9—N1	3.1 (7)	C14—C15—C20—N2	2.0 (7)
C5—C4—C9—N1	−179.9 (4)	C16—C15—C20—N2	178.6 (4)
C3—C4—C9—C8	−172.4 (5)	C14—C15—C20—C19	−173.5 (4)
C5—C4—C9—C8	4.6 (7)	C16—C15—C20—C19	3.2 (7)
